# Investigation Study of Ultrasound Practitioners’ Awareness about Artefacts of Hepatobiliary Imaging in Almadinah Almunawwarah

**DOI:** 10.12669/pjms.38.6.5084

**Published:** 2022

**Authors:** Hassan Ibrahim Alsaedi, Anas Malik Krsoom, Sultan Abdulwadoud Alshoabi, Walaa M. Alsharif

**Affiliations:** 1 Hassan Ibrahim Alsaedi, Department of Diagnostic Radiology Technology, College of Applied Medical Sciences, Taibah University, Almadinah Almunawwarah, Kingdom of Saudi Arabia; 2 Anas Malik Krsoom, Department of Diagnostic Radiology Technology, College of Applied Medical Sciences, Taibah University, Almadinah Almunawwarah, Kingdom of Saudi Arabia; 3 Sultan Abdulwadoud Alshoabi, Department of Diagnostic Radiology Technology, College of Applied Medical Sciences, Taibah University, Almadinah Almunawwarah, Kingdom of Saudi Arabia; 4 Walaa M. Alsharif, Department of Diagnostic Radiology Technology, College of Applied Medical Sciences, Taibah University, Almadinah Almunawwarah, Kingdom of Saudi Arabia

**Keywords:** Acoustic enhancement artefact, Mirror artefact, Acoustic shadowing artefact, Reverberation artefact, Side lobe artefact, Ring down artefact

## Abstract

**Objectives:**

To investigate the knowledge and awareness of ultrasound practitioners’ concerning ultrasound artefacts in evaluating the hepatobiliary system.

**Methods:**

This electronic questionnaire-based comparative study involved the ultrasound practitioners’ who work in the radiology departments in Almadinah Almunawwarah governmental hospitals during the period from 1 November 2020 to 30 April 2021. Spearman’s rho correlation test was used to correlate between knowledge and job, academic qualification, and years of experience. A T-test and cross tabulation test were done to compare the knowledge about artefacts among radiologists and radiologic technologists.

**Results:**

This study involved 94 participants distributed as 22 (23.4%) radiologists and 72 (76.6%) radiologic technologists. The results shows that 85%, 71%, 73%, 69%, 54% and 53% of the participants assigned the acoustic shadowing, acoustic enhancement, ring down, side lobe, reverberation and mirror artefacts, as artefacts respectively. However, 68%, 53%, 19%, 19%, 18%, and 40% of the participants gave correct final diagnosis of acoustic shadowing, acoustic enhancement, ring down, side lobes, reverberation, and mirror artifacts, respectively. Spearman’s rho correlation test shows significant correlation between participants with more than three years experience and knowledge related mirror artefacts (r=0.328, p=0.001). It shows significant correlation between radiologists with knowledge related mirror artefacts (r=0.367, p<0.001). A significant correlation was found between highly qualified participants and knowledge related mirror artefacts (r=0.336, p=0.001) and side lobe artefacts (r=0.237, p=0.008).

**Conclusion:**

The questionnaire-based comparative study of knowledge about artefacts of hepatobiliary ultrasound imaging reveals a high level of Ultrasound practitioners’ knowledge in differentiating artefacts from pathology with a high level of knowledge in identifying hepatobiliary acoustic shadowing and acoustic enhancement artefacts. However, insufficient knowledge was noted in identifying mirror, side lobe, reverberation and ring down artefacts. A direct link was found between academic qualification, years of experience and practioners’ knowledge among.

## INTRODUCTION

Ultrasound is sound waves with frequencies greater than 20 KHz, which is greater than the limits of human hearing with similar terms of travelling to the audible sound. The frequencies, which range from 2 MHz to 18 MHz are used for wide medical purposes. Higher frequencies have smaller wavelengths and can obtain ultrasonograms with smaller details.^1^ Ultrasonography is a diagnostic imaging technique using ultrasound waves to visualise the body structures, including soft tissues, such as liver, muscles, and blood vessels.^1^,^2^ It is a highly valuable imaging modality in assessing liver parenchyma and detecting liver lesions by providing accurate diagnostic information and detecting complications.^3^ It is an ionising radiation free technique, real time, widely available, non-invasive, and low cost.^4^ Ultrasound demands a high level of knowledge and skills, as it is operator dependent, and is less effective in meteorism and obese patients. In addition, deep-seated sub diaphragmatic and very small focal lesions may be overlooked. Lack of knowledge and skills among ultrasound practitioners might influence the quality and safety of the service delivery within radiology departments including Ultrasound units.^4^,^5^

**Fig.1:**
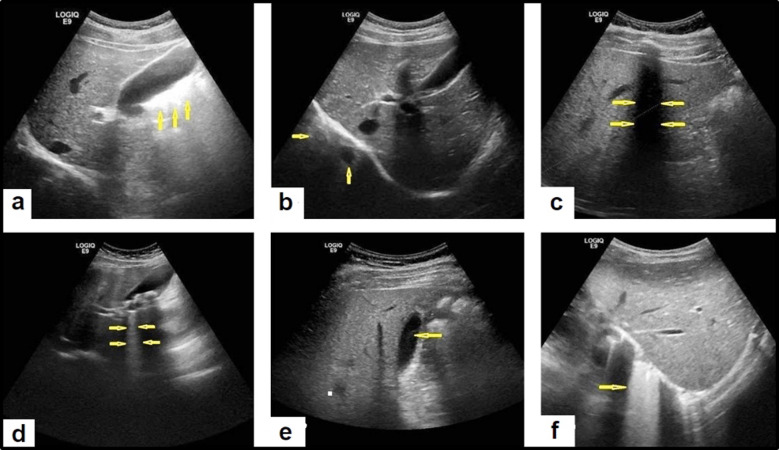
(a) Acountic enhancement artefact appears as a white enhancement area behind the gallbladder. (b) Mirror artefact appears as a duplicated image, equidistant from the right hemidiaphragm but deeper to it. (c) Acoustic shadowing artefact appears as shadow due to strong reduction in the reflected amplitude echo. (d) Reverberation artefact appears as multiple echogenic parallel lines with regular intervals with decreased intensity as the depth increases. (e) Side lobe artefact appears as debris in the gallbladder. (f) Ring down artefact appears as a band or a streak of bands deep to a focus of gas.

Ultrasound image quality is directly linked to factors such as patient position, probe selection, settings of the machine and sonographic window that contribute to image quality.^4^ Artefacts are an alteration in the ultra-sonographic image that does not represent the actual image of the examined anatomic part, and which occur for an array of reasons, due to factors related to dynamic interaction between the Ultrasound beam and the soft tissues or error in technique. The most common artefacts in liver imaging include Acoustic enhancement artefact, Mirror image artefact, Acoustic shadowing artefact, Reverberation artefact, Side lobe artefact, and Ring down artefact^5^ (Fig.1).

The acoustic enhancement artefact occurs as hyperechoic areas on the image, when the Ultrasound beam travels through a tissue with less attenuation than the surrounding tissues, such as fluid-filled structures with weak or no echo reflection.^6^ The mirror image artefact occurs as an image opposite, but equidistant from, the reflective object when the Ultrasound beam passes through a highly reflective non-perpendicular or curved object.^5^,^6^ The acoustic shadowing artefact occurs as shadows, distal to dense reflector structures, when the Ultrasound beam is trying to pass through them.^6^,^7^ The reverberation (Multiple reflection) artefact occurs as multiple regularly spaced duplicated images when the Ultrasound beam passes between highly reflective parallel interfaces.^6^,^7^ The ring down artefact occurs due to reverberation or resonance of Ultrasound beams, when it passes between or within a collection of gas bubbles, such as gas bubbles in the duodenum.^5^-^7^ The side lobe artefact appears as debris in anechoic structures, such as debris in the gallbladder, and occurs because of the peripheral part of the beam, differing in intensity from the main central part and reflected back to the transducer.^7^,^8^

To the authors’ knowledge, there is a notable lack in literature regarding Ultrasound practitioners’ knowledge with a dedicated focus on hepatobiliary artefacts. The aim of the study was, therefore, to investigate the level of knowledge of Ultrasound practitioners regarding distinguishing hepatobiliary artefacts from pathology based on qualifications and years of experience on their knowledge.

## METHODS

### Study Design

A questionnaire-based comparative design was used in this study. An online questionnaire was distributed among radiologists and radiologic technologists in governmental hospitals across Almadinah Almunawwarah city during the period from 1 November 2020 to 30 April 2021 to evaluate their knowledge and skills in identifying hepatobiliary artefacts in Ultrasound imaging. The sample size targeted for this study was 114 Ultrasound practitioners. The calculation was based on the total number of Ultrasound practitioners in Almadinah Almunawwarah city (Approximately=160), with 5% margin of errors and 95% confidence level. Exclusion criteria include Ultrasound practitioners working at private hospitals and centres, and workers who could not be reached. Participation in this study was voluntary, and a total of 94 Ultrasound practitioners agreed to participate. Demographic background information about participants’ gender, jobs, level of academic qualification and years of experience was collected. The questionnaire contains six Ultrasound images which depicted either pathology or artefacts. The datasets were selected from Ultrasound departments in Almadinah Almunawwarah city to represent the range of images typically acquired in the clinical setting. Participants were asked to identify pathology or/and name artefacts in these images.

### Ethical Approval

Governmental hospitals in Almadinah Almunawwarah city were invited to participate in this study. Ethical approval was obtained from the relevant institutional review board (Reference Number: 2020/79/308/DRD). Participants were recruited on a voluntary basis from King Fahad, Almadinah, Ohud, Almeqat, Maternity and children, and Prince Mohammed Bin Abdulaziz hospitals across Almadinah Almunawwarah city.

### Statistical Analysis

The data collected were analysed using the “Statistical Social Sciences Package” (SPSS), version 25, (IBM corp., Armonk, NY), and P-value <0.05 were considered significant. Descriptive variables were presented as frequencies and percentages. Continuous variables were presented as means± standard deviation (SD). The relationship between the various parameters was explained with P-value. A binomial test was used to measure the relationship between categories in different binary variables. Spearman’s rho correlation test was used to show correlation between each of the jobs, qualifications, and years of experience with the knowledge of participants about different artefacts. Paired samples of T-test and Cross-tabulation tests were used to compare the level of knowledge in identifying ultrasound artefacts among radiologists versus radiologic technologists based on level of academic qualification and years of experience.

## RESULTS

This study involved 94 participants. The mean age of the participants was 30.95±9.25. The participants were 52 (55.32%) male and 42 (44.68%) females. The participants were 22 (23.4%) radiologists and 72 (76.6%) radiologic technologists. The participants were distributed as 42 (44.7%) with more than three years of experience and 52 (55.3%) less than three years. Twenty-three (24.4%) of the participants were highly qualified (Ph.D., MD, and MSc). The binomial test shows the relationship between categories in different binary variables. It shows that Ultrasound practitioners were able to differentiate acoustic enhancement artefact, acoustic shadowing, ring down, and side lobes artefacts from pathology (p < 0.001). However, a poor level of knowledge was noted in differentiating mirror artefact (p=0.606), and reverberation artefact (p=0.470), (Table-I).

**Table I:** Binomial test shows the relationship between categories in different binary variables.

Variables	Categories	N	Percentage	P-value
Gender	Male	52	55%	0.353
	Female	42	45%	
Job	Radiologist	22	23%	<0.001
	Radiology Technologist	72	77%	
Qualification	≥MSc	23	24%	<0.001
	≤BSc	71	76%	
Experience	≥3 years	42	45%	0.353
	≤3 years	52	55%	
Acoustic enhancement artefact	Artefact	67	71%	<0.001
	Pathology	27	29%	
Mirror artefact	Artefact	50	53%	0.606
	Pathology	44	47%	
Acoustic shadowing artefact	Artefact	80	85%	<0.001
	Pathology	14	15%	
Reverberation artefact	Artefact	51	54%	0.470
	Pathology	43	46%	
Ring down artefact	Artefact	69	73%	<0.001
	Pathology	25	27%	
Side lobe artefact	Artefact	65	69%	<0.001
	Pathology	29	31%	

Spearman’s rho correlation test between job, academic qualification, and years of experience with the participants’ knowledge concerning different artefacts was done. A significant correlation was found between job (radiologists) and knowledge in identifying mirror artefacts in Ultrasound images (p<0.001). A significant correlation in identifying mirror artefacts and side lobe artefacts was found for qualification. The ability of participants to identify mirror and side lobe artefacts in Ultrasound images was greater for those with a high qualification (p=0.001 and 0.008), respectively. The results also showed a significant correlation between years of experience and knowledge in in identifying mirror artefacts (p=0.001), (Table-II). Paired Samples Test shows a significant variation in knowledge about all artefacts between radiologists and technologists (p<0.001), (Table-III). Paired Samples Test shows a significant variation in knowledge about all artefacts based on years of experience (p<0.001), (Table-IV). Paired Samples Test shows a significant variation in knowledge about all artefacts based on qualification (p<0.001), (Table-V).

**Table II:** Spearman’s rho correlation test between job, certificate, and experience years with the knowledge of participants about different artefacts.

Variables		Acoustic enhancement artefact	Mirror artefact	Acoustic shadowing artefact	Reverberation artefact	Ring down artefact	Side lobe artefact
Job	Correlation Coefficient	-0.073-	-.367-**	0.051	-.104-	-.162-	-.260-*
	P-value	.483	<0.001	.625	.318	.118	0.011
	N	94	94	94	94	94	94
Qualification	Correlation Coefficient	-.252-*	-.336-**	0.040	-0.026-	-.175-	-.273-**
	P-value	0.014	0.001	.702	.804	0.092	0.008
	N	94	94	94	94	94	94
Years of experience	Correlation Coefficient	-.192-	-.328-**	0.045	-.181-	-.154-	-.230-*
	P-value	0.063	0.001	.668	0.081	.140	0.026
	N	94	94	94	94	94	94

**Table III:** Paired Samples Test compared between radiologists and radiologic technologists in knowledge about hepatobiliary artefacts.

Paired variables	Mean	SD	SE	95% Confidence interval of the difference		P-value

				Lower	Upper	
Job × Acoustic enhancement artefact	1.05319	0.64536	0.06656	.92101	1.18537	<0.001
Job × Mirror artefact	1.23404	0.76798	0.07921	1.07674	1.39134	<0.001
Job × Acoustic shadowing artefact	0.91489	0.54199	0.05590	.80388	1.02590	<0.001
Job × Reverberation artefact	1.22340	0.69024	0.07119	1.08203	1.36478	<0.001
Job × Ring down artefact	1.03191	0.66320	0.06840	.89608	1.16775	<0.001
Job × Side lobe artefact	1.07447	0.70694	0.07292	.92967	1.21926	<0.001

**Table IV:** Paired Samples Test compare between more than and less than 3 years of experience in knowledge about hepatobiliary artefact.

Paired variables	Mean	SD	SE	95% Confidence interval of the difference		P-value

				Lower	Upper	
Experience × Acoustic enhancement artefact	0.84043	0.73767	0.07608	0.68934	0.99151	<0.001
Experience × Mirror artefact	1.02128	0.81622	0.08419	0.85410	1.18845	<0.001
Experience × Acoustic shadowing artefact	0.70213	0.60161	0.06205	0.57891	0.82535	<0.001
Experience × Reverberation artefact	1.01064	0.76895	0.07931	0.85314	1.16813	<0.001
Experience × Ring down artefact	0.81915	0.71786	0.07404	0.67212	0.96618	<0.001
Experience × Side lobe artefact	0.86170	0.75635	0.07801	0.70679	1.01662	<0.001

**Table V:** Paired Samples Test compare between high (MD, PhD, and MSc) and low (BSc and Diploma) academic certificates in knowledge about hepatobiliary artefact.

Paired variables	Mean	SD	SE	95% Confidence interval of the difference		P-value

				Lower	Upper	
Certificate × Acoustic enhancement artefact	1.04255	0.70199	0.07240	0.89877	1.18634	<0.001
Certificate × Mirror artefact	1.22340	0.76417	0.07882	1.06689	1.37992	<0.001
Certificate × Acoustic shadowing artefact	0.90426	0.55006	0.05673	0.79159	1.01692	<0.001
Certificate × Reverberation artefact	1.21277	0.66998	0.06910	1.07554	1.34999	<0.001
Certificate × Ring down artefact	1.02128	0.67168	0.06928	0.88370	1.15885	<0.001
Certificate × Side lobe artefact	1.06383	0.71555	0.07380	0.91727	1.21039	<0.001
Certificate × Gain artefact	1.18085	0.63859	0.06587	1.05005	1.31165	<0.001

## Discussion

There was a reference to the need to improve the current education and training of healthcare workers in Saudi Arabia.^9^ This is interesting as, to the researchers’ knowledge, there is no published evidence concerning the level of Ultrasound practitioners’ knowledge in Saudi Arabia. In the context of this study, this study was conducted to evaluate Ultrasound practitioners’ knowledge in identifying hepatobiliary artefacts in Ultrasound images within radiology departments in governmental hospitals across Almadinah Almunawarah city.

In order to facilitate accurate patient diagnosis and treatment, the ultrasonographic imaging artefacts must be recognised and interpreted correctly. Different correction strategies may need to be applied in order to avoid any potential artefacts, such as using multiple imaging planes, changing patient position, using different frequency transducers, or changing configurations to avoid or to minimise the effects of these artefacts.^10^ A significant difference in knowledge concerning different artefacts was found in this study among participants.

The acoustic enhancement artefact occurs as a white enhancement area behind a structure which is caused by a relative increase in intensity of Ultrasound beams when penetrating tissue structures of lower attenuation than the surrounding structures, such as a fluid-filled structure (e.g. gallbladder).^11^ It has diagnostic significance in differentiation of fluid from solid structures that may increase by using tissue harmonic imaging (THI).^7^ In practice, it can be mitigated by changing the angle of the Ultrasound beam while scanning the patient.^12^ In this study, participants (radiologists and radiologic technologists) were able to identfy the acoustic enhancement artefact in Ultrasound images. An association was found in this study between level of academic qualification, years of experience and participants’ level of knowledge in identifying the acoustic enhancement artefact in Ultrasound images. Study participants who have higher levels of academic qualification and years of experience were able to recognise the acoustic enhancement artefact more than others. This was in line with Farajollhi et al. and Andersson et al. who found an association between level of knowledge, academic qualifications, and years of experience.^13^,^14^ However, this was in contrast to Alsharif et al. who found that levels of radiographers’ experience and qualifications did not significantly influence the level of radiographers’ knowledge.^15^

The mirror image artefact appears as a duplicated image, equidistant from the reflective interface but deeper to it. It occurs when the Ultrasound beam passes through a highly reflective non-perpendicular or curved object such as the diaphragm and the echo of the Ultrasound beam reflected back into the transducer.^7^,^12^ This can be avoided in practice by decreasing gain, changing angle of insonation and using multiple imaging windows. It is important to recognise this artefact to avoid mimicking pathology (e.g., diaphragmatic hernia, lung consolidation, or pseudo thickened bowel wall) as it can lead to incorrect diagnosis and cause serious after-effects on a patient’s healthcare and outcomes.^7^,^16^ Quien et al. reported that this artefact can be identified easily, as it appears identical to the original structure in the same frame.^16^ In the current study, radiologists with higher levels of experience revealed high performance in identfying the mirror artefact in Ultrasound images. However, radiologic technologists in this study were not able to distinguish between pathology and the mirror artefact. It seems that radiologists were more familiar with the mirror artefact and pathologic appearances than radiologic technologists.

The acoustic shadowing artefact is the reduction in the reflected amplitude echo caused by reflectors which lie behind structures, which strongly reflect or absorb Ultrasound beams, such as bones, stones or soft-tissue-gas interface. Its clinical importance lies in it confirming diagnosis of stones, calcification and air. It increases with increasing Ultrasound frquency and using THI, and it decreases with increased beam width and using spatial compound imaging.^7^ Knowledge of acoustic shadowing artefacts is necessary to avoid such diagnostic pifalls as diagnosing the caudate lobe of the liver as liver pathology or pancreatic pseudocyst.^17^ This artefact can by minimised by changing angles and positions of the patients.^17^,^18^ The study participants were able to identify the acoustic shadowing artefact. There was a slight difference in knowledge between radiologists and radiologic technologists based on levels of academic qualifications and years of experience concerning this artefact. Ability to recognise the acoustic shadowing artefact by radiologic technologists may reflect a good understanding of the physics principle regarding this artefact or their familiarity with this artefact on their daily clinical practice.

The reverberation artefact occurs when Ultrasound echoes reflected repeatedly between highly reflective interfaces in parallel.^5^,^7^ It appears as multiple echogenic parallel lines with regular intervals with decreased intensity as the depth increases.^19^ It is mimicking debris in cystic structures, which is an undesirable artefact except in rare cases such as detecting the presence of abnormal air. This artefact can be reduced by THI, decreasing gain, changing angle of insonation (AOI) and using multiple windows.^7^ A poor knowledge was found among radiologists and radiologic technologists in distinguishing between reverberation artefacts and pathology, and poor knowledge about the appearance of this artefact was also noted. The results reflect the random distribution of the answers, and no significant differences in identifying the reverberation artefact were found for years of experience and level of academic qualification. This result was similarly found by Alsharif et al. and Foley et al.^15^,^20^ This gap in knowledge about the reverbation artefact may be attributed to the rare occurrence of this artefact. Regrettably, there were no previous studies to compare with our results.

The ring down artefact appears as a band or a streak of bands deep to a focus of gas.^19^ It occurs due to resonance vibrations within gas bubbles, and, clinically, it can be Ultrasoundeful in determining abnormal foci of gas, such as portal venous gas, abscess, pneumoperitoneum, pneumobilia and emphysematous infections and may be reduced by using spatial compound imaging.^7^,^19^ Despite the significance of knowledge about this artefact, this study found a poor level of knowledge among the study participants regarding the ring down artefact. This artefact was falsely diagnosed as pathology, acoustic enhancement artefact, or acoustic shadowing artefact. This artefact was correctly identified by radiologists who have a high level of academic qualifications and years of experience.

The side lobes (secondary lobe) artefact appears as debris in an anechoic structure like the gallbladder, due to reflected echoes coming back from Ultrasound waves transmitted outside the main Ultrasound beam.^19^ It can be corrected or reduced by using THI, reducing the gain, changing AOI, using multiple windows or advanced transducer design.^7^,^19^ Ilovitsh et al. introduces a new optically-inspired method to improve contrast of Ultrasound image by decreasing the side lobe artefact without reducing the frame rate or the resolution.^21^ Knowledge about this artefact is mandatory to avoid the pitfall of sludge diagnosis.

This study shows a significant difference between radiologists and radiologic technologists in distinguishing side lobe artefacts. It was surprising to find that radiologists who have a high level of academic qualifications and years of experience were not able recognise the side lobe artefact from other gallbladder diseases, such as the sludge that may mimic it in appearance. This reason may reflect that the side lobes artefact can only occur in old machines, which are rarely used nowadays. As providing accurate diagnoses falls under the radiologists’ responsibility, it is crucial that radiologists be familiar with pathological changes and grasp the impact of different artefacts on image quality.

There was a lack of published researches and this caused difficulties in comparing the study’s findings; however, some of the more relevant studies were identified. Therefore, further research in these areas is recommended to focus on this critical topic to avoid diagnostic pitfalls, either in the hepatobiliary system or other body parts in Ultrasound images.

### Limitations

A limitation of this study is that it was based on an online questionnaire due to the COVID-19 pandemic so, the Ultrasound images were displayed on different devices of the participants with wide variations in resolution. Furthermore, the fundamental trade-off between the features of their devices (display) may still limit the ability to determine their true efficiency to distinguish artifacts from pathology in ultrasound imaging. Also, it must be mentioned that the number of radiologists in Almadinah Almunawwarah is few, and access to them is difficult in most cases.

## Conclusions

A high level of Ultrasound practitioners’ knowledge in differentiating artefacts from pathology, identifying hepatobiliary acoustic shadowing and acoustic enhancement artefacts was found. However, insuffiecient knowledge was noted among Ultrasound practitioners in identifing mirror, side lobe, reverberation, and ring down artefacts. In addition, a low level of radiologic technologists’ knowledge, comparing with radiologists, in identifing the mirror, side lobes, and ring down artefacts was found, which is of concern. High level of academic qualifications and years of experience made a difference in the ability to identify image artefacts except for the acoustic shadowing and ring artefacts.

### Author’s Contribution

**HIA** **&** **AMK:** Collected and analyzed data. **SAA:** Prepared the final draft of the manuscript. **WMA:** Revised the manuscript and improved language. All authors have critically reviewed and approved the final draft and are responsible for the content and similarity index of the manuscript.
